# Abstract of the Proceedings of the Buffalo Medical Association

**Published:** 1877-05

**Authors:** 


ART. III.—*Abstract of the Proceedings of the Buffalo Medical Association.*

TUESDAY EVENING, February 5, 1877.

The President, Dr. WYCKOFF, in the Chair.

On motion, Dr. LUCIEN HOWE was appointed Secretary *pro tem.*

The minutes of the January meeting were read and adopted.

The applications of Drs. F. J. BAKER and J. A. PETIT for membership were received.

In reply to a question from Dr. BRIGGS, Dr. SAMO reviewed briefly the case presented by him at the January meeting stating that he did not think the case was one of disease of the brain; that the iodide potass was given in two-grain doses three times daily.

Dr. MYNTER related a case of stricture of the œsophagus in a man aged 67. The symptoms first presented themselves three or four years ago. He first came under Dr. Mynter's care the latter part of November ; could then only take food in a liquid form. Electrolysis was proposed, but objected to. In December he could not swallow fluids even. He then tried electrolysis, and succeeded in dilating the passage to such an extent that he could swallow with considerable ease both fluid and solid food. He has increased in weight at the rate of a pound per week. Ten elements of the zinc carbon battery were used. The stricture is again contracting, and the question now presents itself: Shall the number of elements be increased ? The stricture is near the cardiac orifice.

Dr. Cronyn said cases of this nature are to be treated in accordance with the cause. When a syphilitic origin is suspected pot. iodid, in large doses, is of benefit. In reference to the dose, he thought that in numerous syphilitic sequelæ large doses were indicated.

Dr. S. S. Green cited the case of a man to whom he administered gr. x at a dose ; after taking two doses he had neuralgic pains, coryza, paralysis of the muscles of the throat, etc. On discontinuing the medicine the symptoms ceased. The medicine was again resumed, and the symptoms returned. In another case a sort of ptyalism was induced.

Dr. Barnes reported a case of strangulated hernia in a woman aged 70. An operation was made, the stricture relieved, and the sac and contents returned entire. The Doctor left town three days after, but Dr. Van Peyma informs him that the patient continued vomiting for three days, and that on the third day it became stercoraceous ; but finally she recovered. He did not know whether it was from an incarceration, which persisted after the reduction, or whether there was paralysis of the intestine.

Dr. Cronyn said he saw a case of strangulated femoral hernia in a woman which, after two hours' persistent taxis, was returned. At the end of twenty-four hours the patient was easy, but she died on the third day. He thought that sloughing of the intestine had been produced from long continued strangulation. A delicate question arises as to how long to wait before resorting to an operation. He thinks that the operation is often made too late.

Dr. Rochester asked if anyone present had used the aspirator to reduce the size of the hernial protrusion before resorting to taxis.

Dr. W. W. Miner said that he had not seen hernia reduced by aid of the aspirator, but had recently had to do with a case at the Sisters' Hospital, in which a collection of fluid apparently in the sac outside the intestine of an irreducible hernia, was removed with a capillary trocar, two or three weeks after the descent of the hernia. The fluid was of about eight ounces in quantity, clear, straw-colored. It reaccumulatd to the same amount or near, by the second day after, then diminished, was tapped after ten days, with an ordinary trocar, and a scanty dark fluid escaped. A tumor the size of a goose egg had suddenly appeared at the site of a long standing inguinal hernia; a hard mass remained, but after six weeks of disability the patient returned to work.

Dr. E. R. Barnes was appointed essayest, with Dr. F. W. Bartlett alternate.

On motion the Association adjourned.

---

Tuesday Evening, March 6, 1877.

In the absence of the President, Dr. J. F. Miner was chosen Chairman *pro tem.*

The minutes of the February meeting were read and adopted.

Dr. Hopkins moved that the Secretary be instructed to cast the ballot of the Association for Drs. F. J. Baker and J. H. Pettit, who were proposed at the February meeting. They were accordingly elected.

Dr. E. R. Barnes read a paper upon the "Hip-Joint." (See Art. II., this number.

Dr. Brush mentioned a point referred to by Dr. Allis, in the *Philadelphia Medical Times* for September, 1876, in regard to the diagnostic value of the fascia lata in fracture of the cervix femoris. The point was briefly this, That in a sound person standing erect, with his limbs resting symmetrically on the floor, the fascia lata offered a firm resistence to the examination of the head of the great trochanter. If the neck of the femur be fractured, the muscles which render the femoral aponeurosis tense have no firm point of support. And there is no resistence to an examination of the trochanter.

Dr. Hopkins asked if there was any direct evidence that the muscular support relieved the head of the femur in sustaining the direct weight of the body.

Dr. Barnes said he did not know of any conclusive evidence on that point.

Dr. Miner being called away said he had been very much interested in Dr. Barnes' paper, and would like to be able to make some remarks upon it. Before leaving the chair, therefore, he would appoint Dr. Barnes to continue the subject at the next meeting.

Dr. W. W. Miner asked as to the serviceability of the limb after fracture of the neck of the femur.

Dr. Rochester said that the results would naturally follow in proportion to age. He related a case of bony union, which though a departure from the subject was of interest. A lady 91 or 92 years of age fractured the tibia and fibula by a fall. She was so old that she had lost her mind, and was completely helpless. Splints were applied simply as a retentative apparatus, with no idea that union would result; but, to his surprise, perfect bony union occurred in about the ordinary time..

Dr. Howe presented a report of his examination of the eyes of school children. (Dr. Howe's paper is crowded out of the Journal this month, but has appeared in the daily papers.)

Dr. Rochester said a paper from Dr. Howe should be placed to some use; that it should be put in print and placed before the teachers, either through the Superintendent's office or otherwise. He said he had occasion, several years ago, to refer to the public schools and their miserable hygienic condition.

Dr. Lothrop said that the paper of Dr. Howe had been of much interest. That in the present condition of the school department the Superintendent had but little to say or to do in the matter. He moved that the paper be requested for publication, and that it be furnished to the public press. He thought that the paper should be presented to the public in this manner.

Dr. Hopkins said that he rose to second the motion, and he desired to refer to the fact that the paper illustrated the feasibility of the Association conducting inquiries in the line of public sanitary affairs.

Under "Prevailing Diseases," influenza and mumps were reported.

DR. ROCHESTER said that he had seen several cases of mumps, also some children with enlargement of the glands about the neck other than those involved in true mumps. He had also seen a great deal of severe influenza. Two of his cases had proved fatal, both dying suddenly. One was 64 years old, on the morning of the day of his death. The patient, pretty well at 2 P. M., began to grow cold, pale and faint, and at 5 P. M. died. The other, a child aged two, was sick four or five days, but not considered seriously so. At 10 P. M. the breathing was a little labored, growing gradually worse. At 1 A. M. he was sent for, but before he could reach the child's bedside it was dead. He thought the probability was that this disease was not only epidemic but communicable. This latter statement he knew it was difficult to prove, but his observations had led him to believe it true.

On motion the Association adjourned.

